# Immunization with an adenovirus-vectored TB vaccine containing Ag85A-Mtb32 effectively alleviates allergic asthma

**DOI:** 10.1007/s00109-017-1614-5

**Published:** 2018-01-04

**Authors:** Yiling Zhang, Ying Feng, Liang Li, Xianmiao Ye, Jinlin Wang, Qian Wang, Pingchao Li, Na Li, Xuehua Zheng, Xiang Gao, Chufang Li, Feng Li, Baoqing Sun, Kefang Lai, Zhong Su, Nanshan Zhong, Ling Chen, Liqiang Feng

**Affiliations:** 1 State Key Laboratory of Respiratory Disease, The First Affiliated Hospital of Guangzhou Medical University, Guangzhou, China; 2 Guangzhou Institutes of Biomedicine and Health, Chinese Academy of Sciences, 190 Kai Yuan Avenue, Science Park, Guangzhou, China; 3 Department of Respiratory Medicine, Guizhou Provincial People’s Hospital, Guiyang, China; 4 Institute of Infectious Diseases, Guangzhou Eighth People’s Hospital, Guangzhou Medical University, Guangzhou, China

**Keywords:** Adenoviral vector, Allergic asthma, Cytokines, Immunization, Mycobacterial antigens

## Abstract

**Abstract:**

Current treatments for allergic asthma primarily ameliorate symptoms rather than inhibit disease progression. Regulating the excessive T helper type 2 (Th2) responses may prevent asthma exacerbation. In this study, we investigated the protective effects of Ad5-gsgAM, an adenovirus vector carrying two mycobacterial antigens Ag85A and Mtb32, against allergic asthma. Using an ovalbumin (OVA)-induced asthmatic mouse model, we found that Ad5-gsgAM elicited much more Th1-biased CD4^+^T and CD8^+^T cells than bacillus Calmette-Guérin (BCG). After OVA challenge, Ad5-gsgAM-immunized mice showed significantly lowered airway inflammation in comparison with mice immunized with or without BCG. Total serum immunoglobulin E and pulmonary inducible-nitric-oxide-synthase were efficiently reduced. The cytokine profiles in bronchial-alveolar-lavage-fluids (BALFs) were also modulated, as evidenced by the increased level of interferon-γ (IFN-γ) and the decreased level of interleukin (IL)-4, IL-5, and IL-13. Anti-inflammatory cytokine IL-10 was sharply increased, whereas pro-inflammatory cytokine IL-33 was significantly decreased. Importantly, exogenous IL-33 abrogated the protective effects of Ad5-gsgAM, revealing that the suppression of IL-33/ST2 axis substantially contributed to protection against allergic inflammation. Moreover, regulatory T cells were essential for regulating aberrant Th2 responses as well as IL-33/ST2 axis. These results suggested that modulating the IL-33/ST2 axis via adenovirus-vectored mycobacterial antigen vaccination may provide clinical benefits in allergic inflammatory airways disease.

**Key messages:**

•Ad5-gsgAM elicits Th1 responses and suppresses Th2-mediated allergic asthma in mice.

•Ad5-gsgAM inhibits IL-33/ST2 axis by reducing IL-33 secretion but not ILC2 recruiting.

•Treg is essential for modulating Th2 responses and IL-33/ST2 axis by Ad5-gsgAM.

**Electronic supplementary material:**

The online version of this article (10.1007/s00109-017-1614-5) contains supplementary material, which is available to authorized users.

## Introduction

Asthma is a complex syndrome characterized by airway hyperresponsiveness (AHR), airflow obstruction, and airway infiltration of inflammatory cells, which affects 5–16% of people worldwide [1]. The treatments available mainly depend on inhaled corticosteroids and sometimes in combination with long-acting beta agonists [1]. These treatments, which are usually administrated as life-long daily medications, only control symptoms but not inhibit disease progression [1]. Long-term use of these drugs leads to resistance and side effects, especially in patients with severe asthma. Recently, blocking antibodies against immunoglobulin E (IgE), interleukin (IL)-4, IL-5, IL-13, and IL-17 have emerged as adjunct therapies [2, 3]. However, a single antibody only shows moderate, if any, benefits in patients with severe asthma or in particular subgroups [3]. Adverse effects such as immuno-suppression are also observed [2, 4, 5]. Novel approaches, which can reduce and even prevent asthma exacerbation, are urgently needed.

T helper type 2 (Th2) cytokines including IL-4, IL-5, and IL-13 play crucial roles in the trigger and progression of allergic asthma, whereas Th1 cytokines such as interferon-γ (IFN-γ) and anti-inflammatory cytokine IL-10 ameliorate aberrant Th2 responses [6]. Th1-biased immune responses elicited by bacillus Calmette-Guérin (BCG) regulate excessive Th2 responses and alleviate airway inflammation in asthmatic animal models [7–9]. However, whether BCG confers protective effects in asthmatic patients remains controversial [10, 11]. Although BCG vaccination is inversely correlated to atopic diseases according to several epidemiological studies [12, 13], its benefits for asthma are absent in several clinical trials [10, 11]. The underlying mechanism remains unclear, but the variable capacity of different BCG strains in inducing Th1 responses may be an explanation [14, 15]. Mycobacterial antigens such as Ag85B or Ag85A-IL-17A fusion delivered as DNA or purified proteins were explored as immuno-therapeutics for allergic asthma [16, 17]. Nevertheless, the capacity of plasmid DNA or purified protein in inducing Th1-biased responses is relatively limited [18]. Alternative strategies, such as utilizing adenoviral vector to carry mycobacterial antigens, may generate enhanced Th1 responses and thereby provide consistent protection.

Replication-incompetent adenoviral vectors are able to elicit high levels of Th1 and CD8^+^T cell responses. Currently, adenoviral-vectored tuberculosis (TB) vaccines have shown great promise in preclinical trials and are undergoing clinical trials [19, 20]. Previously, we reported a recombinant adenovirus type 5 carrying two immuno-dominant mycobacterial antigens Ag85A and Mtb32 (Ad5-gsgAM), which induced robust systemic and pulmonary cellular responses [19]. Interestingly, Ag85A mainly raised IFN-γ-producing CD4^+^T cells, whereas Mtb32 predominantly elicited CD8^+^T cells which also secreted IFN-γ [19]. Both Th1 CD4^+^T cells and type 1 (Tc1) CD8^+^T cells may contribute to the suppression of Th2 responses, eosinophilia, as well as IgE production [21]. The cytokines secreted by these cells, especially IFN-γ, also participate in controlling Th2 responses and allergy [22]. We proposed that, if Ad5-gsgAM generates high levels of Th1- and Tc1-biased responses following allergen exposure, it may modulate Th2 responses and efficiently suppress allergic asthma.

In this study, we evaluated the protective effects of Ad5-gsgAM in an ovalbumin (OVA)-induced asthmatic mouse model. The effects of Ad5-gsgAM immunization on AHR, pulmonary inflammation, and Th1/Th2 responses in the airway were studied. The underlying mechanisms were also analyzed.

## Materials and methods

### Animals and asthma models

Six-week-old female C57BL/6 mice were purchased and housed in a specific pathogen-free facility in the Experimental Animals Center of Guangzhou Institutes of Biomedicine and Health (GIBH). The protocols of animal experiments were approved by the Institutional Animal Care and Use Committee of GIBH (Permit No. 2013026).

The asthma model was established as described previously [8, 16]. In brief, mice were sensitized by three intraperitoneal (I.P.) injections at weekly intervals with 50 μg of chicken OVA (Grade V, Sigma-Aldrich, St. Louis, MO) in 2 mg of alum (Sigma-Aldrich, St. Louis, MO). Three weeks after the final sensitization, the mice were challenged with aerosolized OVA (2% in saline) for 40 min for three consecutive days. One day after the last challenge, the mice were evaluated for AHR and then sacrificed.

### Replication-incompetent adenovirus and immunization

Ad5 empty vector (Ad5) and Ad5-gsgAM were described in our previous study [19]. One week after the final OVA sensitization, the mice were immunized with saline (termed OVA mice), Ad5 (2.5 × 10^9^ viral particles per mouse; termed OVA/Ad5 mice), or Ad5-gsgAM (2.5 × 10^9^ viral particles per mouse; termed OVA/gsgAM mice) by intramuscular (I.M.) inoculation (*n* = 8 to 10 mice per group). One week later, the animals were intranasally (I.N.) boosted with the respective agents mentioned above. As for BCG (2 × 10^5^ colony forming units per mouse; termed OVA/BCG mice) immunization, twice subcutaneous (S.C.) administrations were performed at similar time points to Ad5-gsgAM immunization. Additional mice treated with saline at each time point were used as healthy controls (termed Saline mice). To investigate the effects of Ag5-gsgAM on IL-33/ST2 axis, OVA/gsgAM mice were I.N. inoculated with recombinant mouse IL-33 (mIL-33; R&D Systems, Abingdon, UK) at 0.5 μg per mouse at 6 and 2 days before challenge. To investigate the roles of regulatory T cells (Tregs), OVA/gsgAM mice were I.P. inoculated with rat anti-mouse CD25 monoclonal antibody (PC61, 200 μg per mouse; ThermoFisher Scientific, Waltham, MA) for depletion of CD25^+^ Tregs at 6 and 2 days before challenge. Additional mice treated with rat anti-mouse IgG1 (R&D Systems, Abingdon, UK) were used as isotype controls. Each animal experiment was performed three times independently.

### Analysis of airway allergic inflammation

The measurement of AHR, the collection and analysis of cells in the BALFs, and the examination of lung tissue sections are described in Supporting Information.

### Enzyme-linked immunosorbent assay

The IgE concentration in the sera and the cytokine concentrations in the sera or BALFs were measured using enzyme-linked immunosorbent assay (ELISA) kits according to the manufacturer’s instructions. ELISA kits for murine IgE, IL-4, IL-5, and IL-10 were purchased from BD Bioscience (San Diego, CA). ELISA kits for murine IL-13, IL-33, ST2, IFN-γ, and TNF-α were purchased from R&D Systems (Abingdon, UK).

### Enzyme-linked immunospot assay

IFN-γ- and IL-4-releasing cells are examined using enzyme-linked immunospot assay (ELISpot) assays as described in Supporting Information.

### Immunohistochemistry

The immunostaining of iNOS was performed as previously reported [23]. In brief, the lung tissue sections were deparaffinized for 20 min in xylene, dehydrated for 10 min in 100% ethanol, and washed with PBS for 10 min. The endogenous peroxidase activity was then inhibited with 0.3% H_2_O_2_ for 15 min. Finally, the sections were incubated with a rat anti-iNOS antibody (Abcam, UK) overnight at 4 °C, developed with a goat anti-rat IgG, and revealed using immuno-peroxidase kit (Santa Cruz, Dallas, TX). The quantification of iNOS-expressing cells was described in Supporting Information.

### Intracellular cytokine staining (ICS) and flow cytometry

Intracellular cytokine staining (ICS) assays were performed as previously described [19]. In brief, splenocytes and lung lymphocytes were isolated and seeded into 96-well plates, incubated with peptide pools of Ag85A or Mtb32 (2 μg/ml per peptide) or with 40 ng/ml Phorbol 12-myristate-13-acetate (PMA) and 1000 ng/ml ionomycin (Sigma-Aldrich, St. Louis, MO). One hour later, brefeldin A (10 μg/ml, BD Biosciences, San Diego, CA) was added and the PMA+ionomycin-stimulated cells were incubated for additional 5 h, whereas the peptide pool-stimulated cells were incubated for additional 10 h. The cells were harvested and stained with surface antibodies (CD3-PerCP, CD4-FITC, CD8-APC; BD Biosciences, San Diego, CA) for 1 h and were then washed, permeabilized, and stained with intracellular antibodies (IFN-γ-PE; BD Biosciences, San Diego, CA). Finally, the cells were detected with a BD Accuri™ C6 instrument.

To assess Tregs in the spleen and mediastinal lymph nodes (MLN), lymphocytes were isolated and stained with surface antibodies (CD3-Pacific Blue, CD4-FITC, CD25-APC; BD Biosciences, San Diego, CA) for 1 h and then washed, permeabilized, and stained with intracellular antibody (FoxP3-PE; BD Biosciences, San Diego, CA). The cells were detected with a BD Accuri™ C6 instrument.

### Western blot analysis

The expression of iNOS in the lung tissues was evaluated through Western blot analysis as described in Supporting Information.

### Data analysis and statistics

The flow cytometry data were collected and analyzed using the FlowJo software (version 7.6.2; Tree Star, Inc., Ashland, OR). The gel graphs were analyzed using ImageJ software (NIH, Bethesda, MD). The data were presented as the mean ± standard error of mean (SEM). The statistical comparisons between groups were analyzed by one-way analysis of variance (ANOVA), and Bonferroni post hoc tests were performed when multiple groups were compared. All the calculations were conducted with SPSS version 13.0 (SPSS Inc., Chicago, IL), and *P* values < 0.05 were considered statistically significant.

## Results

### Ad5-gsgAM immunization elicits robust Th1 CD4^+^ and Tc1 CD8^+^ T cell responses in OVA-sensitized mice

**Fig. 1:**
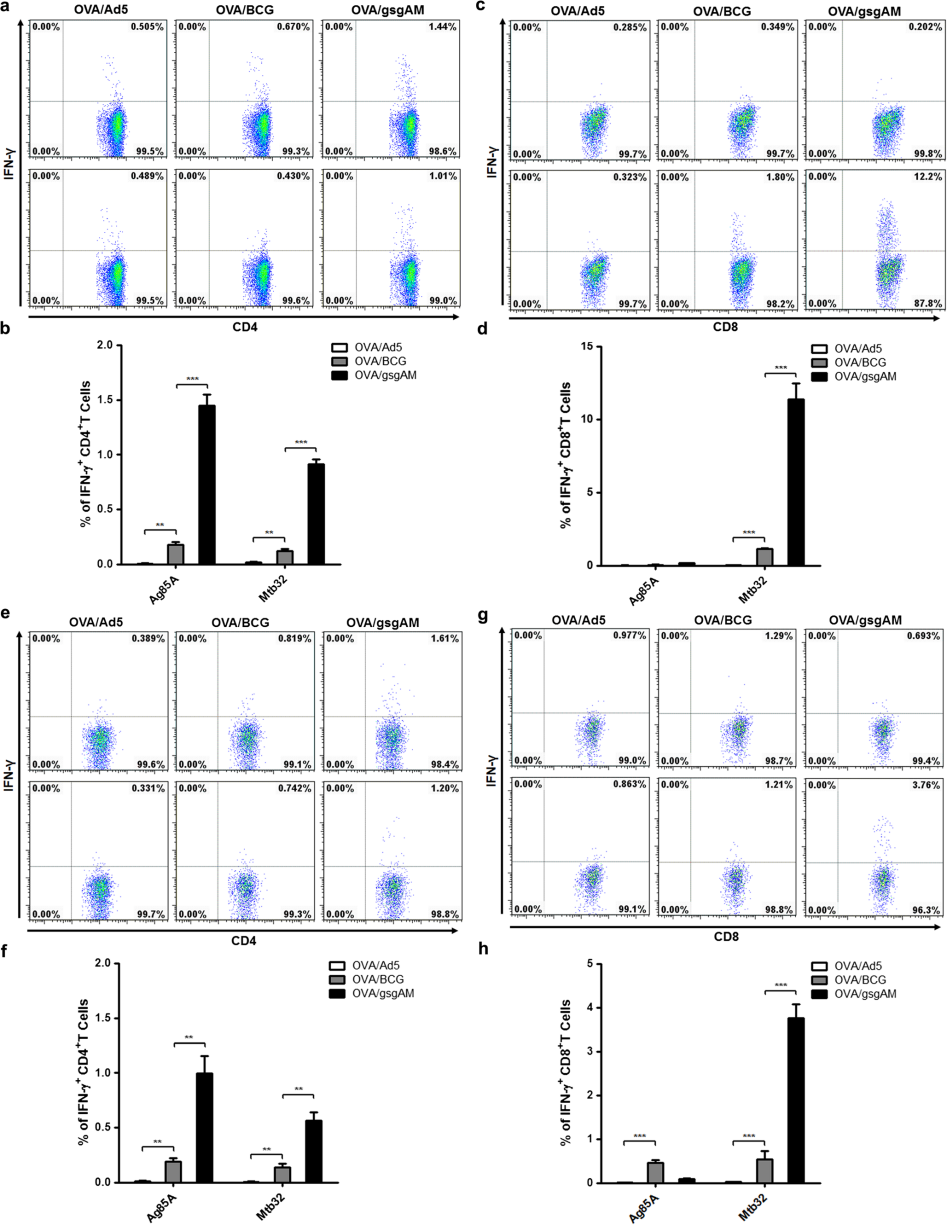
Ad5-gsgAM immunization generates robust antigen-specific T cell responses in OVA-sensitized mice. OVA-sensitized mice were immunized as depicted in Fig. S1. One week after the final immunization, lymphocytes were isolated from the spleens and lungs of different groups of mice and were stimulated with peptide pools of Ag85A or Mtb32. Unstimulated lymphocytes from each group were used as controls. Then, the cells were stained with CD3-PerCP, CD4-FITC, CD8-APC, and IFN-γ-PE and subjected to FACS analysis. **a** Representative dot plots of Ag85A-specific (upper panel) and Mtb32-specific (bottom panel) CD4^+^T cells secreting IFN-γ in the spleens. **b** The percentages of antigen-specific IFN-γ^+^CD4^+^T cells in total CD4^+^T cells in the spleens. **c** Representative dot plots of Ag85A-specific (upper panel) and Mtb32-specific (bottom panel) CD8^+^T cells secreting IFN-γ in the spleens. **d** The percentages of antigen-specific IFN-γ^+^CD8^+^T cells in total CD8^+^T cells in the spleens. **e** Representative dot plots of Ag85A-specific (upper panel) and Mtb32-specific (bottom panel) CD4^+^T cells secreting IFN-γ in the lungs. **f** The percentages of antigen-specific IFN-γ^+^CD4^+^T cells in total CD4^+^T cells in the lungs. **g** Representative dot plots of Ag85A-specific (upper panel) and Mtb32-specific (bottom panel) CD8^+^T cells secreting IFN-γ in the lungs. **h** The percentages of antigen-specific IFN-γ^+^CD8^+^T cells in total CD8^+^T cells in the lungs. Data are presented as mean ± standard error of the mean (SEM, *n* = 5 mice per group). **P < 0.01, ***P < 0.001. Similar results were obtained from two additional experiments

To assess the cellular responses elicited by Ad5-gsgAM following allergen sensitization, we administered Ad5-gsgAM, BCG, or Ad5 to OVA-sensitized C57BL/6 mice (Fig. S1a, b). Both BCG and Ad5-gsgAM elicited significant Ag85A-specific and Mtb32-specific IFN-γ^+^CD4^+^T and Mtb32-specific IFN-γ^+^CD8^+^T cell responses in the spleen (Fig. 1a–d). Notably, the frequencies of IFN-γ^+^CD4^+^T and IFN-γ^+^CD8^+^T cells were much higher in Ad5-gsgAM-immunized mice than in BCG-immunized mice (Fig. 1a–d), suggesting that Ag85A and Mtb32 have strong immunogenicity when harbored in an adenovirus vector. Similar to what we observed in non-asthmatic mice [19], Ag85A predominantly induced Th1 CD4^+^T cell responses, whereas Mtb32 mainly generated CD8^+^T cell responses (Fig. 1a–d). Pulmonary cellular responses exhibited similar trends as splenic ones (Fig. 1e–h). Moreover, when stimulated with PMA and ionomycin, much more IFN-γ-producing CD4^+^T and CD8^+^T cells were observed in Ad5-gsgAM-immunized mice than in BCG- or Ad5-immunized mice (Fig. S1c–f). Together, Ad5-gsgAM elicits much stronger Th1 CD4^+^T and Tc1 CD8^+^T cell responses than BCG in OVA-sensitized mice.

### Ad5-gsgAM immunization reduces AHR, eosinophilia, and total serum IgE in OVA-induced asthmatic mice

**Fig. 2:**
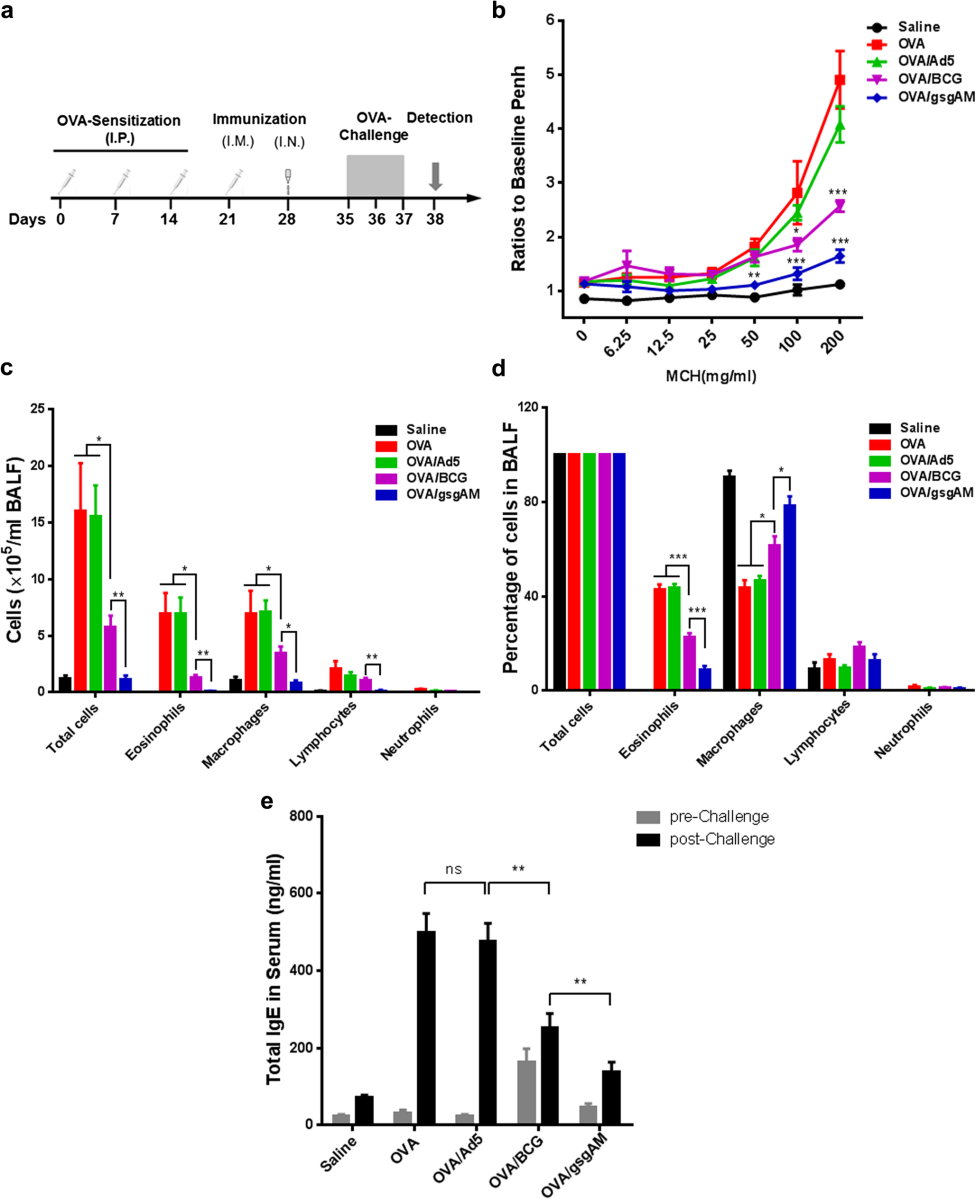
The effects of Ad5-gsgAM immunization on AHR, inflammatory cell infiltration, and total serum IgE. One week after sensitization with saline or OVA, mice were immunized with saline, Ad5, BCG, or Ad5-gsgAM and then challenged with OVA for three consecutive days. Twenty-four hours after the final challenge, they were subjected to whole-body plethysmograph analysis followed by sacrifice and lavage. **a** The schedules of sensitization, immunization, challenge, and detection. **b** The AHR in response to increased doses of inhaled MCh. **c** The absolute numbers of total cells, eosinophils, macrophages, lymphocytes, and neutrophils in the BALFs were counted using H&E staining. **d** The percentages of total cells, eosinophils, macrophages, lymphocytes, and neutrophils in the BALFs. **e** The concentrations of total serum IgE before and after challenge were assessed by ELISA. Data are presented as the mean ± SEM (*n* = 5 mice per group). Representative results from one of three independent experiments are shown. *P < 0.05, **P < 0.01, ***P < 0.001, ns, no significance

We administrated Ad5-gsgAM, BCG, or Ad5 to OVA-sensitized mice before OVA challenge (Fig. 2a). OVA/gsgAM but not OVA/Ad5 or OVA/BCG mice showed significantly lower Penh values in response to 50 mg/ml aerosolized MCh, in comparison with OVA mice (Fig. 2b). Ad5-gsgAM immunization led to a sharp reduction at both 100 and 200 mg/ml of MCh in comparison with Ad5 immunization, whereas BCG immunization only resulted in a moderate decrease of Penh values at 100 mg/ml of MCh and a significant decrease at 200 mg/ml of MCh (Fig. 2b). Thus, Ad5-gsgAM inhibits AHR more efficiently than BCG.

Next, we analyzed the number and type of cells in the BALFs. Both Ad5-gsgAM and BCG significantly reduced the numbers of total cells, eosinophils, and macrophages as compared to Ad5 (Fig. 2c). Notably, Ad5-gsgAM immunization resulted in a greater reduction than BCG immunization (Fig. 2c), suggesting that Ad5-gsgAM has greater potency in suppressing inflammatory cell infiltration. The percentage of eosinophils was also much lower in OVA/gsgAM mice than in other experimental mice (Fig. 2d). Intriguingly, in the BALFs of OVA/gsgAM mice, macrophages were the dominant cells, similar to that of Saline mice (Fig. 2d). Moreover, Ad5-gsgAM but not BCG immunization decreased the number of lymphocytes (Fig. 2c). Together, Ad5-gsgAM potentially inhibits the infiltration of inflammatory cells.

We also examined the total serum IgE. Before OVA challenge, comparable serum IgE was detected for all groups but OVA/BCG mice, for which we observed a higher IgE level (Fig. 2e). After challenge, OVA and OVA/Ad5 but not OVA/BCG mice showed enhancement in IgE levels, whereas OVA/gsgAM mice displayed a significantly lower level of serum IgE than OVA/BCG mice (Fig. 2e), suggesting that Ad5-gsgAM suppresses the production of IgE more efficiently than BCG.

### Ad5-gsgAM immunization ameliorates pulmonary inflammation and mucus over-production

**Fig. 3:**
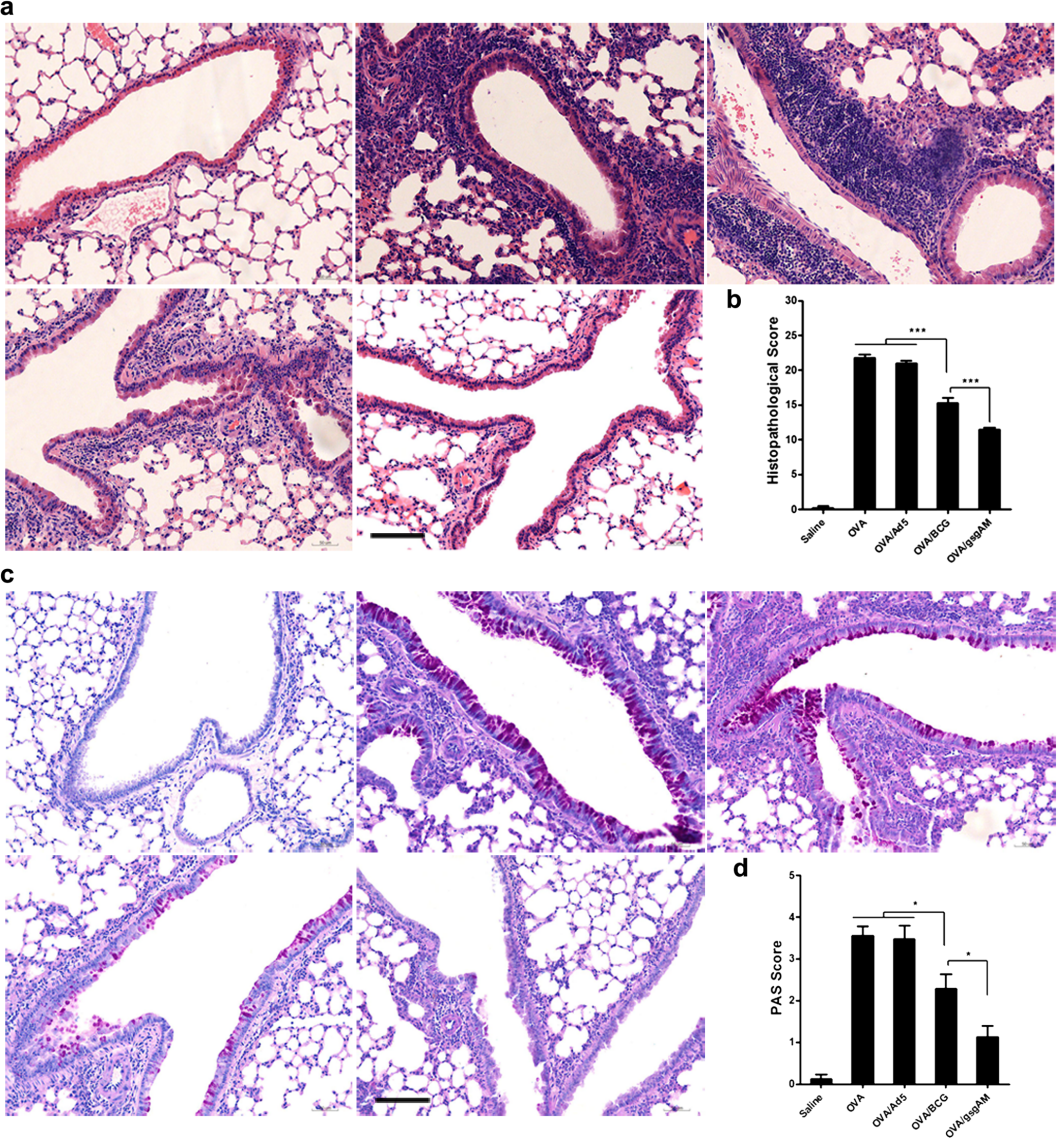
The effects of Ad5-gsgAM immunization on airway inflammation and goblet cell hyperplasia. Twenty-four hours after the final challenge, mouse lung tissue sections were subjected to histological examination. **a** Representative photomicrographs of H&E-stained lung sections from each group of mice (upper left panel: Saline mice; upper middle panel: OVA mice; upper right panel: OVA/Ad5 mice; bottom left panel: OVA/BCG mice; bottom middle panel: OVA/gsgAM mice). Bar = 100 μm. **b** Quantitative assessment of the peribronchial and perivascular inflammation. **c** Representative photomicrographs of PAS-stained lung sections from each group of mice (upper left panel: Saline mice; upper middle panel: OVA mice; upper right panel: OVA/Ad5 mice; bottom left panel: OVA/BCG mice; bottom middle panel: OVA/gsgAM mice). Bar = 100 μm. **d** Quantitative assessment of goblet cell hyperplasia. Data are presented as the mean ± SEM (*n* = 5 mice per group). Representative results from one of three independent experiments are shown. *P < 0.05, ***P < 0.001

We then analyzed the lung tissue sections using H&E and PAS staining. The peribronchial and perivascular inflammation in OVA/gsgAM and OVA/BCG mice was attenuated as compared to OVA or OVA/Ad5 mice (Fig. 3a, b). Importantly, Ad5-gsgAM immunization showed a further alleviation in comparison with BCG immunization (Fig. 3a, b). Consistent with these observations, Ad5-gsgAM immunization remarkably reduced mucus-secreting goblet cells, whereas BCG immunization only achieved moderate reduction (Fig. 3c, d). These results suggested that Ad5-gsgAM substantially reduces airway inflammation and mucus over-production upon allergen challenge.

### Ad5-gsgAM immunization suppresses the expression of iNOS in the airway

**Fig. 4:**
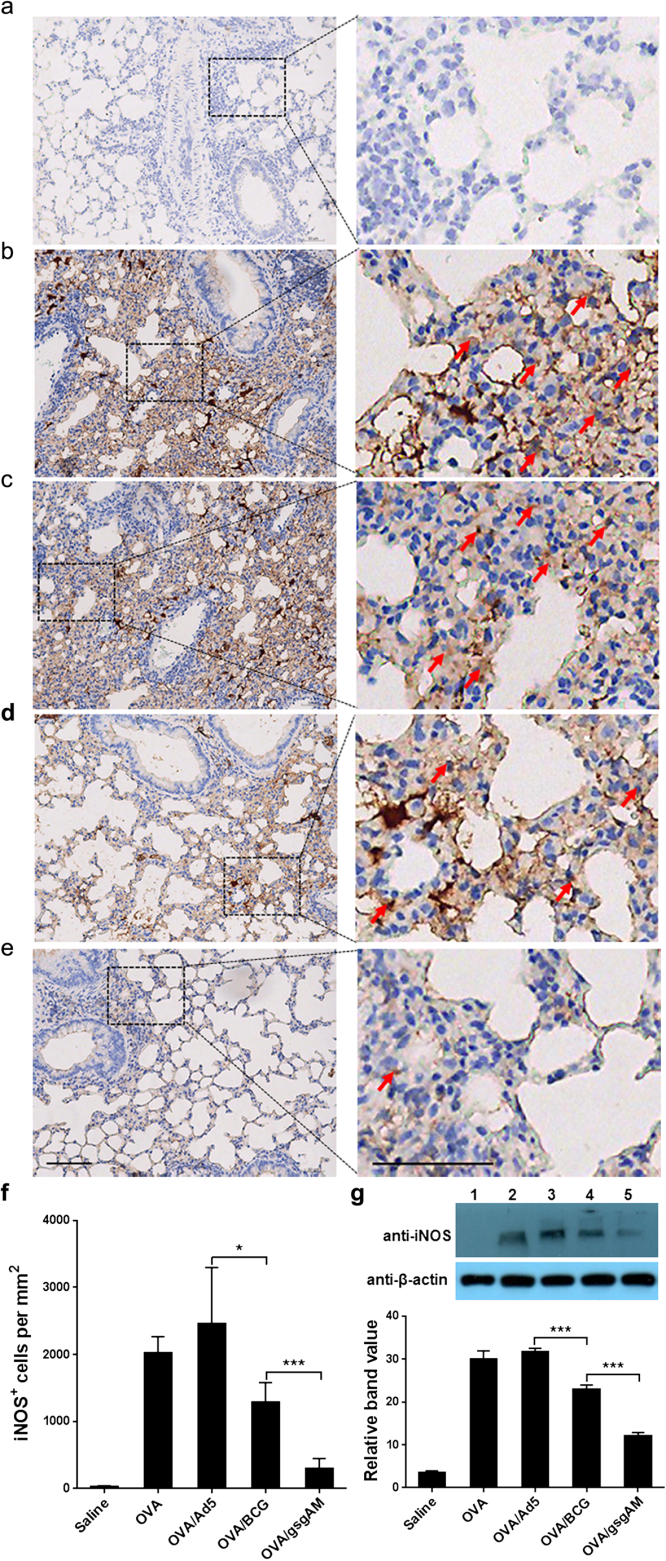
The expression of iNOS in the lungs of the experimental and control mice. Twenty-four hours after the final challenge, mouse lung tissue sections were immuno-stained with anti-iNOS antibodies. **a**–**e** Representative photomicrographs of immuno-stained lung sections from Saline (**a**), OVA (**b**), OVA/Ad5 (**c**), OVA/BCG (**d**), and OVA/gsgAM (**e**) mice were shown. The iNOS-expressing cells are marked by red arrows. Bar = 100 μm. **f** Quantitative analysis of the iNOS-expressing cells in the lung tissue sections from each group of mice. Three sections of each mouse were analyzed. The iNOS-expressing cells in five random fields of each section were counted and calculated as cell numbers per square millimeter. Data are presented as the mean ± SEM (*n* = 4 to 6 mice per group). Representative results from one of three independent experiments are shown. *P < 0.05, ***P < 0.001. **g** The concentration of iNOS in the lung tissue homogenates pooled from five mice per group were examined with Western blot analysis (upper panel) and then quantified using ImageJ software (bottom panel). One representative gel from three independent experiments was shown. Lane 1: Saline mice; Lane 2: OVA mice; Lane 3: OVA/Ad5 mice; Lane 4: OVA/BCG mice; Lane 5: OVA/gsgAM mice. The density value of each band was read out and the net band value was obtained by deducting the background from the band value. The relative band value was calculated as the ratio of the net band value of iNOS vs that of β-actin. Data are presented as the mean ± SEM (*n* = 5 mice per group). Representative results from one of three independent experiments are shown. *P < 0.05, ***P < 0.001

Because NO has been reported to promote the pathophysiology of asthma [24], we analyzed whether Ad5-gsgAM immunization affected the expression of iNOS. A large number of iNOS-expressing cells were observed in OVA-challenged mice treated with or without Ad5 but not in healthy control mice (Fig. 4a–c, f). Ad5-gsgAM significantly decreased the number of iNOS-expressing cells, whereas BCG resulted in a moderate reduction (Fig. 4d–f). Consistently, the amount of iNOS in OVA/gsgAM mice was reduced in comparison with OVA, OVA/Ad5, and even OVA/BCG mice (Fig. 4g), revealing that Ad5-gsgAM inhibits the expression of iNOS with greater efficiency than BCG.

### Ad5-gsgAM immunization modulates the excessive pulmonary Th2 cytokines and suppresses IL-33/ST2 axis

**Fig. 5:**
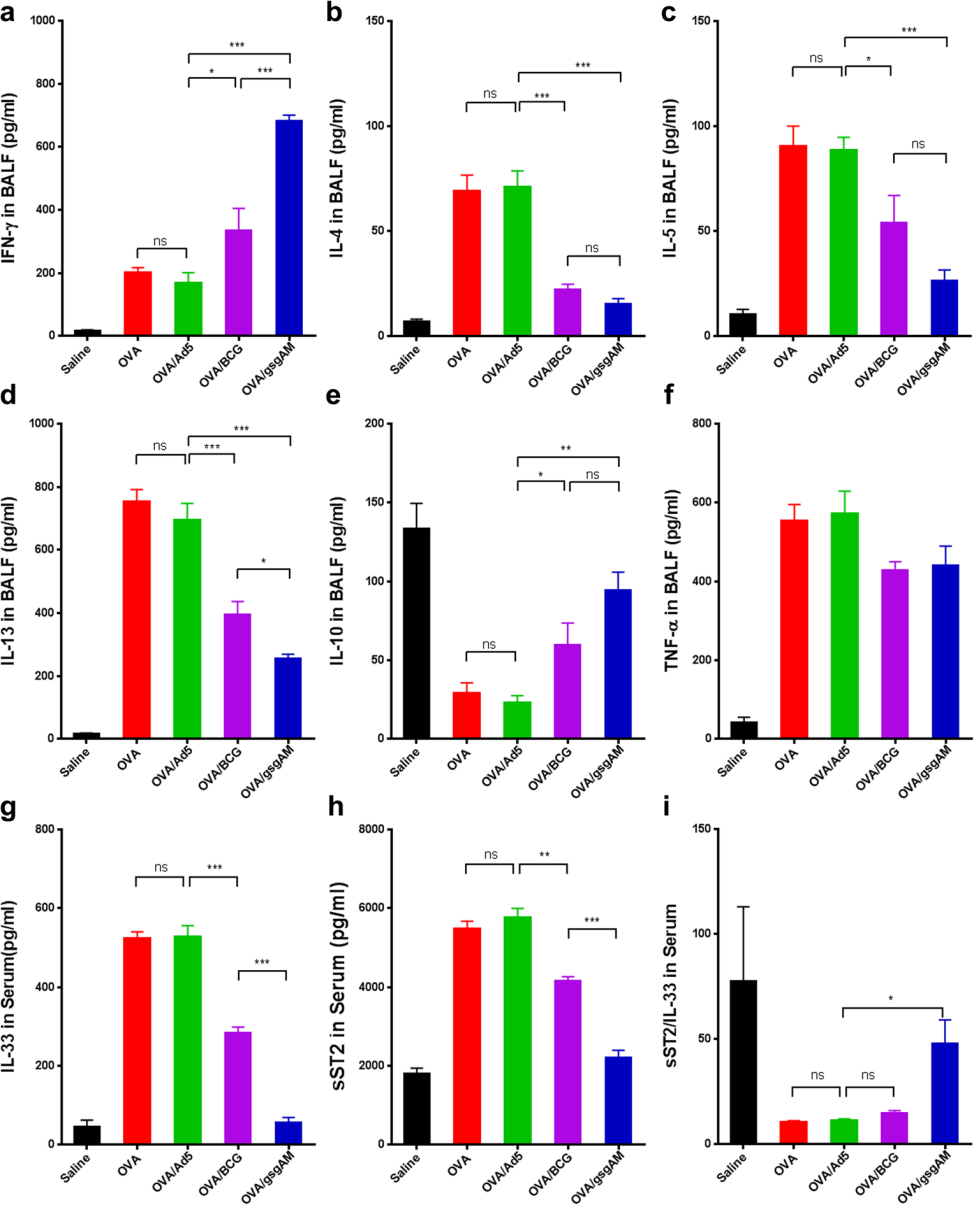
Profiling of cytokines in the BALFs and serums. Twenty-four hours after the final challenge, the cytokines in the BALFs and serums of different group of mice were evaluated by ELISA. **a**–**f** The concentrations of IFN-γ (**a**), IL-4(**b**), IL-5 (**c**), IL-13 (**d**), IL-10 (**e**), and TNF-α (**f**) in the BALFs. **g**–**i** The contents of IL-33 (**g**) and sST2 (**f)** in the serums were assessed by ELISA and the ratios (**i**) of sST2 to IL-33 were calculated and shown. Data are presented as the mean ± SEM (*n* = 5 mice per group). Representative results from one of three independent experiments are shown. *P < 0.05, **P < 0.01, ***P < 0.001. ns, no significance

To determine whether Ad5-gsgAM immunization modulates Th1/Th2 immune responses, we assessed the cytokine profiles in the BALFs. OVA/gsgAM mice showed significantly elevated level of IFN-γ compared to OVA/BCG mice (Fig. 5a), and both were significantly higher in comparison with OVA/Ad5 mice (Fig. 5a), suggesting that Ad5-gsgAM immunization enhances pulmonary Th1 cytokines. On the other hand, both Ad5-gsgAM and BCG immunization sharply reduced the contents of IL-4, IL-5, and IL-13 (Fig. 5b–d). Notably, Ad5-gsgAM immunization resulted in significantly lower level of IL-13 in comparison with BCG immunization (Fig. 5d). IL-10, an anti-inflammatory cytokine mainly secreted by Tregs and monocytes [25], was significantly elevated in OVA/gsgAM and OVA/BCG mice (Fig. 5e). TNF-α, however, were comparable in experimental animals (Fig. 5f). Together, Ad5-gsgAM enhances Th1 and anti-inflammatory cytokines but decreases Th2 cytokines in the airway.

IL-33/ST2 axis promotes the expression of pro-inflammatory cytokines and accumulation of inflammatory cells [26]. We therefore investigated whether Ad5-gsgAM affected this pathway. Both Ad5-gsgAM and BCG decreased serum IL-33 as compared to Ad5 (Fig. 5g), with OVA/gsgAM mice displaying a further reduction than OVA/BCG mice (Fig. 5g). OVA/gsgAM and OVA/BCG mice also showed significantly lower levels of soluble ST2 (sST2) (Fig. 5h). Interestingly, the ratio of sST2 to IL-33 was higher in OVA/gsgAM mice than in OVA or OVA/Ad5 mice (Fig. 5i). Because sST2 competitively blocks the binding of IL-33 to ST2 [27], Ad5-gsgAM immunization efficiently modulates IL-33/ST2 axis.

### CD4^+^T and CD8^+^T cells elicited by Ad5-gsgAM suppress airway inflammation.

To confirm the protective effects of Ad5-gsgAM-induced T cell responses, we performed adoptive transfer assays. CD4^+^T and CD8^+^T cells were isolated from the spleens and lungs of Ad5-gsgAM- or Ad5-immunized mice. One day before challenge, OVA-sensitized mice were inoculated with splenic and lung T cells through intravenous and intranasal routes, respectively (Fig. S2a). Both CD4^+^T and CD8^+^T cells from Ad5-gsgAM- but not Ad5-immunized mice significantly reduced eosinophilia (Fig. S2b, c). The suppressive efficacy of CD4^+^T cells is comparable to CD8^+^T cells. Consistently, IL-5, IL-13, IL-33, as well as sST2 were reduced, as observed in OVA/gsgAM mice (Fig. S2d–g). Therefore, CD4^+^T and CD8^+^T cells induced by Ad5-gsgAM have protective effects against allergic inflammation.

### Exogenous IL-33 abolishes the anti-asthmatic effects of Ad5-gsgAM.

**Fig. 6:**
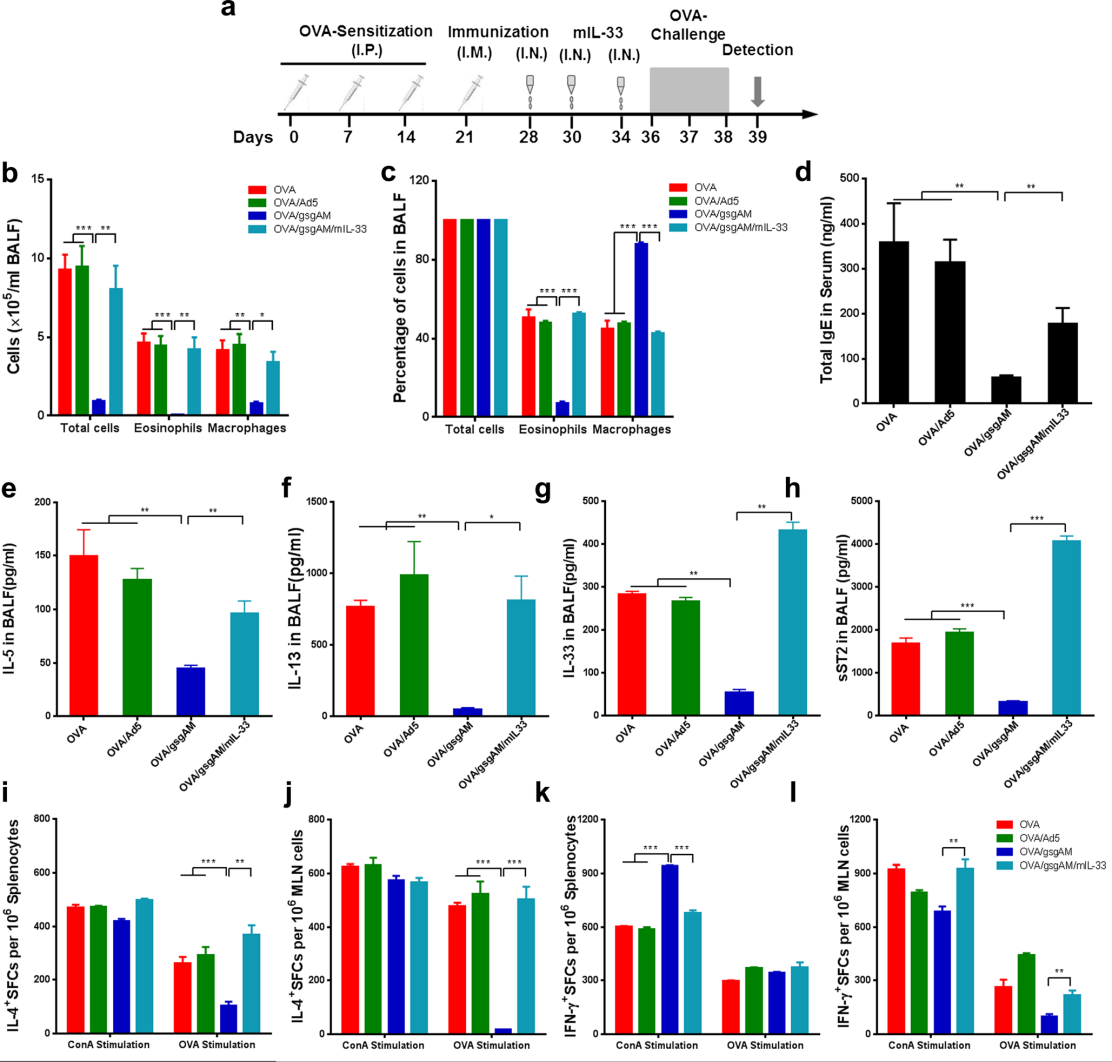
Exogenous IL-33 reverses the suppressive effects of Ad5-gsgAM on asthma. Six-week-old mice were sensitized with OVA, immunized with Ad5 or Ad5-gsgAM, inoculated with saline or mouse IL-33 (termed as OVA/gsgAM/mIL-33), and then challenged with OVA. Twenty-four hours after the final challenge, the mice were sacrificed. **a** Schedules of OVA sensitization, immunization, IL-33 inoculation, OVA challenge, and detection. **b** The absolute numbers of total cells, eosinophils, and macrophages in the BALFs were counted based on H&E staining. **c** The percentages of total cells, eosinophils, and macrophages in the BALFs. **d** The contents of IgE in the serums of the experimental mice were determined by ELISA. **e**–**h** The contents of IL-5 (**e**), IL-13 (**f**), IL-33 (**g**), and sST2 (**h**) in the BALFs were determined by ELISA. **i** The IL-4-secreting cells in the splenocytes. **j** The IL-4-secreting cells in the MLN lymphocytes. **k** The IFN-γ-secreting cells in the splenocytes. **l** The IFN-γ-secreting cells in the MLN lymphocytes. Lymphocytes from the spleens and MLNs were stimulated with 10 μg/ml ConA or 10 μg/ml OVA. After a 24-h incubation for IFN-γ or a 48-h incubation for IL-4 detection, the IL-4- or IFN-γ-secreting cells were assessed by ELISpot. Data are presented as the mean ± SEM (*n* = 5 mice per group). Representative results from one of three independent experiments are shown. *P < 0.05, **P < 0.01, ***P < 0.001

To further investigate the modulation of IL-33/ST2 axis by Ad5-gsgAM, we administrated mIL-33 to Ad5-gsgAM-immunized mice prior to OVA challenge (Fig. 6a). Exogenous mIL-33 significantly elevated inflammatory cell infiltration into airways (Fig. 6b, c). In addition, mIL-33 partially restored the total serum IgE (Fig. 6d). The modulation of aberrant Th2 cytokines by Ad5-gsgAM also disappeared in the presence of mIL-33, as IL-5 and IL-13 in the BALFs substantially increased (Fig. 6e–g). Notably, exposure to mIL-33 induced a higher level of sST2 in the BALFs (Fig. 6h). Therefore, exogenous inoculation with mIL-33 abolishes the protective effects of Ad5-gsgAM.

We also assessed the alterations of cellular responses after mIL-33 inoculation. Comparable IL-4-producing cells were observed in the spleen and MLN when stimulated with ConA (Fig. 6i, j). However, mIL-33 significantly enhanced OVA-specific IL-4-secreting cells, both in the spleen and MLN (Fig. 6i, j). Meanwhile, mIL-33 reduced ConA-stimulated IFN-γ-secreting cells in the spleen (Fig. 6k) but enhanced ConA- and OVA-stimulated IFN-γ-secreting cells in the MLN (Fig. 6l). These results suggested that exogenous mIL-33 alters the IL-4^+^ and IFN-γ^+^ cellular responses both in the spleen and in the airway.

We then examined group 2 innate lymphoid cells (ILC2s), one of the target cells of IL-33, in the airways of asthmatic mice treated with Ad5-gsgAM or Ad5. The frequency of ILC2 is comparable in these two groups of mice (Fig. S3), suggesting that Ad5-gsgAM treatment does not decrease the number of pulmonary ILC2s. Thus, Ad5-gsgAM modulates IL-33/ST2 pathways more likely through decreasing the production of IL-33 but not the recruitment of ILC2.

### Suppression of pulmonary inflammation by Ad5-gsgAM is dependent on regulatory T cells

**Fig. 7:**
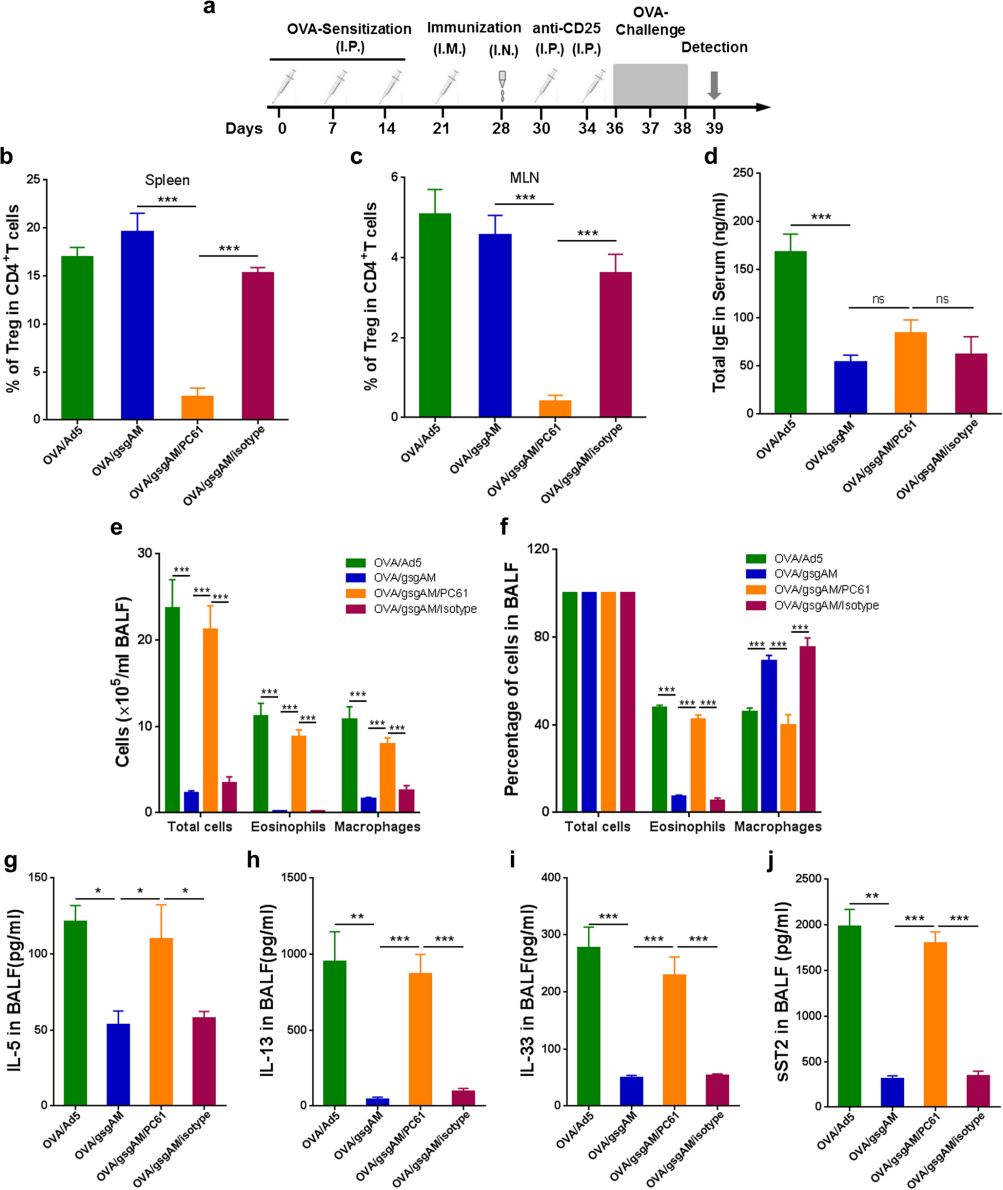
Treg is essential for the suppressive effects of Ad5-gsgAM on asthma. Six-week-old mice were sensitized with OVA, immunized with Ad5 or Ad5-gsgAM, inoculated with anti-CD25 antibody PC61 or isotype control (termed as OVA/gsgAM/PC61 or OVA/gsgAM/isotype, respectively), and then challenged with OVA. Twenty-four hours after the final challenge, the mice were sacrificed. **a** Schedules of OVA sensitization, immunization, antibody inoculation, OVA challenge, and detection. **b**, **c** The frequency of CD4^+^CD25^+^FoxP3^+^ cells in the spleen (**b**) and MLN (**c**) were examined by flow cytometry. **d** The contents of IgE in the serums were determined by ELISA. **e** The absolute numbers of total cells, eosinophils, and macrophages in the BALFs were counted based on H&E staining. **f** The percentages of total cells, eosinophils, and macrophages in the BALFs. **g**–**j** The contents of IL-5 (**g**), IL-13 (**h**), IL-33 (**i**), and sST2 (**j**) in the BALFs were determined by ELISA. Data are presented as the mean ± SEM (*n* = 5 to 7 mice per group). Representative results from one of three independent experiments are shown. *P < 0.05, **P < 0.01, ***P < 0.001. ns, no significance

To investigate whether Tregs participant in the protective effects of Ad5-gsgAM, we depleted CD4^+^CD25^+^FoxP3^+^ cells with anti-CD25 antibody PC61 before challenge (Fig. 7a). Most Tregs were depleted in PC61-treated but not isotype-treated mice (Fig. 7b, c). Treg depletion did not reverse the suppression of serum IgE by Ad5-gsgAM (Fig. 7d) but enhanced inflammatory cells, especially eosinophils, in the BALFs (Fig. 7e, f), implying that Tregs were essential for suppressing inflammatory cell infiltration but not IgE production. IL-5 and IL-13 were significantly elevated (Fig. 7g, h). The content of IL-33 and sST2 was also sharply increased in Treg-depleted animals (Fig. 7i, j), suggesting that Tregs were important for the modulation of IL-33/ST2 axis. Together, Tregs are essential for suppressing airway inflammation by Ad5-gsgAM, but there are other pathways participating in the inhibition of IgE production.

## Discussion

To our knowledge, we are the first to adopt Ad5, the most potent vector for generating T cell responses [28], as the carrier of mycobacterial antigens for the purpose of preventing allergic asthma. Ad5-gsgAM elicits much stronger T cell responses, including Th1 CD4^+^T and Tc1 CD8^+^T cells, than BCG (Figs. 1and S1). After OVA challenge, Ad5-gsgAM provides efficient protection against allergic asthma (Figs. 2 and 3). Ad5-gsgAM not only inhibits iNOS expression and modulates excessive Th2 responses (Figs. 4 and 5) but also suppresses the IL-33/ST2 axis through inhibiting IL-33 production (Figs. 6, S2, and S3). Moreover, Tregs are essential for the protective effects of Ad5-gsgAM (Fig. 7). Our results support that Ad5-gsgAM is worthy for further exploration as an alternative immunotherapy against asthma.

Ad5-gsgAM has at least three advantages over other candidates such as BCG and mycobacterial proteins: (i) Ag85A and Mtb32 harbored in Ad5 vector have strong immunogenicity (Figs. 1 and S1). Both Th1 CD4^+^T and Tc1 CD8^+^T cells induced by Ag85A and Mtb32 may downregulate Th2 responses and have benefits for allergic asthma (Fig. S2) [21]. More than 90% decrease of BALF cells was observed in Ad5-gsgAM-immunized mice (Fig. 2), whereas treatment with pMG-Ad85B or Ag85a-IL-17A only achieved 37% or nearly 40% decrease of BALF cells, respectively [16, 17], demonstrating the robustness of Ad5-gsgAM. Thus, Ad5-gsgAM may evade the variant efficiency of different BCG strains and provide consistent protection [14, 15]. (ii) Incorporating two immuno-dominant antigens may cover more T cell epitopes than those containing only a single antigen and thereby facilitates to generate immune responses in genetically heterologous individuals [29]. (iii) Recombinant Ad5 vectors, including those carrying mycobacterial antigens, have good safety profiles [18, 20], which may allay the safety concerns of BCG, especially for immuno-suppressed individuals.

In accordance with the inhibition of airway inflammation, Ad5-gsgAM suppresses the production of total serum IgE (Fig. 2), similar to another study using Ag85B protein [30]. Surprisingly, BCG immunization elicits significant serum IgE before OVA challenge (Fig. 2). Actually, an early study showed that BCG elicited higher level of total IgE than a modified BCG in mice [31]. Individuals exposed to *Mycobacterium tuberculosis* or *Mycobacterium avium* Subsp. *Paratuberculosis* infection generated IgE responses [32, 33]. Although mycobacterial antigen is generally considered to downregulate IgE production [34], our and others’ results implied that some mycobacterium including BCG could elicit IgE responses. Therefore, Ad5-gsgAM may be better than BCG in controlling IgE production.

iNOS and its product nitric oxide (NO) play important roles in tissue damage during airway inflammation [35]. Selective inhibitors of iNOS reduce the influx of inflammatory cells in animals [23]. Although these inhibitors have shown minimal benefits for asthma in clinical trials, knocking-out all of the NOS isoforms decreases airway inflammation and reduces Th2 cytokines such as IL-4, IL-5, and IL-13 in asthmatic mice [24]. Thus, the suppression of iNOS by Ad5-gsgAM may contribute to the inhibition on airway inflammation and Th2 responses (Fig. 4).

The mechanism by which BCG or mycobacterial antigens alleviate asthma remains unclear. According to the “hygiene hypothesis,” Th1 responses antagonize excessive Th2 responses and prevent the onset of allergic asthma [6]. IFN-γ^+^T cells other than Tregs contribute to the protective effects in asthmatic mice receiving neonatal BCG immunization [36]. However, other studies indicate that IL-10-secreting Tregs generated by BCG or freeze-dried BCG are important for the suppression of allergic inflammation [37]. We showed that Ad5-gsgAM-induced CD4^+^T and CD8^+^T cells were protective against airway inflammation (Fig. S2). We also showed that Tregs were essential for the modulation of Th2 responses and airway inflammation, because IL-10 in the airways were sharply increased in OVA/gsgAM mice (Fig. 5), whereas Tregs depletion reversed the inhibition of airway inflammation (Fig. 7). Therefore, multiple pathways, including Th1 responses and Tregs, participate in the modulation of Th2 responses by Ad5-gsgAM.

Recently, the importance of IL-33/ST2 axis has been recognized in the trigger and maintenance of allergic asthma [38]. IL-33 is an “alarmin” and releases upon cell injury [39, 40], whereas ST2 has two isoforms, the full-length transmembrane ST2 and the truncated soluble sST2 [41]. The interaction of IL-33 with ST2 activates ILC2, memory Th2, and mast cells, which secrete IL-5 and IL-13 and facilitate pulmonary eosinophilic inflammation [26, 42]. sST2 is a decoy receptor of IL-33 and competitively inhibits IL-33/ST2 pathway [41]. The ratio of IL-33 vs sST2 reflects the bioavailability of circulating IL-33 and the activity of IL-33/ST2 pathway [43–45]. Ad5-gsgAM efficiently reduced serum and pulmonary IL-33, and elevated the ratio of sST2 vs IL-33 to a similar level to healthy control mice, reflecting an efficient suppression on IL-33/ST2 pathway (Figs. 5 and 6). Exogenous mIL-33 abolished the protective effects of Ad5-gsgAM, consistent with others’ results [46]. Interestingly, exogenous IL-33 induced large amounts of sST2 (Fig. 6), possibly because sST2 could be synthesized by mast cells when activated by IL-33 [44]. The reduction of serum and pulmonary IL-33 in Ad5-gsgAM-treated mice may limit the secretion of sST2 (Figs. 5 and 6). However, Ad5-gsgAM did not reduce the frequency of pulmonary ILC2 (Fig. S3). Therefore, Ad5-gsgAM modulates IL-33/ST2 axis more likely through reducing IL-33 production but not decreasing pulmonary ILC2.

Several limitations still existed in this study. Firstly, we used non-invasive whole-body plethysmography (Penh) to measure AHR (Fig. 2). Compared to lung resistance (*R*_L_) measurement, Penh has significant advantages such as no need of anesthesia and surgery and thereby avoids the influence on physiological parameters of experimental animals [47]. However, the sensitivity of Penh in analyzing pulmonary mechanics is relatively limited [48, 49]. Both Penh and *R*_L_ detected AHR in severe asthma, but only *R*_L_ detected AHR in mild asthma [47]. Nevertheless, Ad5-gsgAM significantly decreased AHR when measured by Penh, consistent to the results of serum IgE and airway inflammation (Figs. 2 and 3). Therefore, although more accurate results may be dependent on *R*_L_ measurements, Penh detection may also give insightful results. Secondly, further studies are needed to dissect the link between innate and adaptive immunity elicited by Ad5-gsgAM during the protection against allergic asthma. Thirdly, the function mechanisms of Ad5-gsgAM other than modulation of IL-33/ST2 axis need to be clarified using IL-33^−/−^ or ST2^−/−^ mice, which is unavailable in our laboratory.

In summary, we demonstrated that Ad5-gsgAM generates robust Th1 and Tc1 responses and inhibits airway inflammation in an OVA-induced asthmatic mouse model. The modulation of excessive Th2 responses and IL-33/ST2 pathway and the suppression of iNOS expression contribute to the protective effects. Tregs are essential for the protective effects conferred by Ad5-gsgAM. Our results provide new insights for the design of alternative therapeutic vaccines against allergic asthma.

## Electronic supplementary material

ESM 1 (PDF 218 kb)

ESM 2 (PDF 158 kb)

ESM 3 (PDF 112 kb)

ESM 4 (PDF 130 kb)
