# Supplementary material for: Immunization with an adenovirus-vectored TB vaccine containing Ag85A-Mtb32 effectively alleviates allergic asthma
Source: J Mol Med (Berl). 2018 Jan 4;96(3):249–63. doi: 10.1007/s00109-017-1614-5 (PMC5859035; doi:10.1007/s00109-017-1614-5)
Supplement: Supplementary file 1 — (PDF 218 kb) [file 109_2017_1614_MOESM1_ESM.pdf]

Title: Immunization with an adenovirus-vectored TB vaccine containing Ag85A-Mtb32 effectively alleviates allergic asthma

Journal Name: Journal of Molecular Medicine

Authors: Yiling Zhang[1,2,3¶], Ying Feng[1,2¶], Liang Li[1,2¶], Xianmiao Ye[2], Jinlin Wang[2], Qian Wang[2], Pingchao Li[1,2], Na Li[1], Xuehua Zheng[2], Xiang, Gao[2], Chufang Li[1], Feng Li[4], Baoqing Sun[1], Kefang Lai[1], Zhong Su[2], Nanshan Zhong[1], Ling Chen[1,2]*, and Liqiang Feng[2]*

Affiliations:

[1]State Key Laboratory of Respiratory Disease, The First Affiliated Hospital of Guangzhou Medical University, Guangzhou, China, [2]Guangzhou Institutes of Biomedicine and Health, Chinese Academy of Sciences, Guangzhou, China, [3]Department of Respiratory Medicine, Guizhou Provincial People's Hospital, Guiyang, China. [4]Institute of Infectious Diseases, Guangzhou Eighth people's Hospital, Guangzhou Medical University, China.

[¶]These authors contributed equally to this work.

*Correspondence should be addressed to:

L.F. (E-mail: feng_liqiang@gibh.ac.cn; Tel: +86-20-32015289; Fax: +86-20-32015299), or L.C. (E-mail: chen_ling@gibh.ac.cn; Tel: +86-20-32015289; Fax: +86-20-32015299), Guangzhou Institutes of Biomedicine and Health, Chinese Academy of Sciences, 190 Kai Yuan Avenue, Science Park, Guangzhou, China.

Supplementary Figure S1

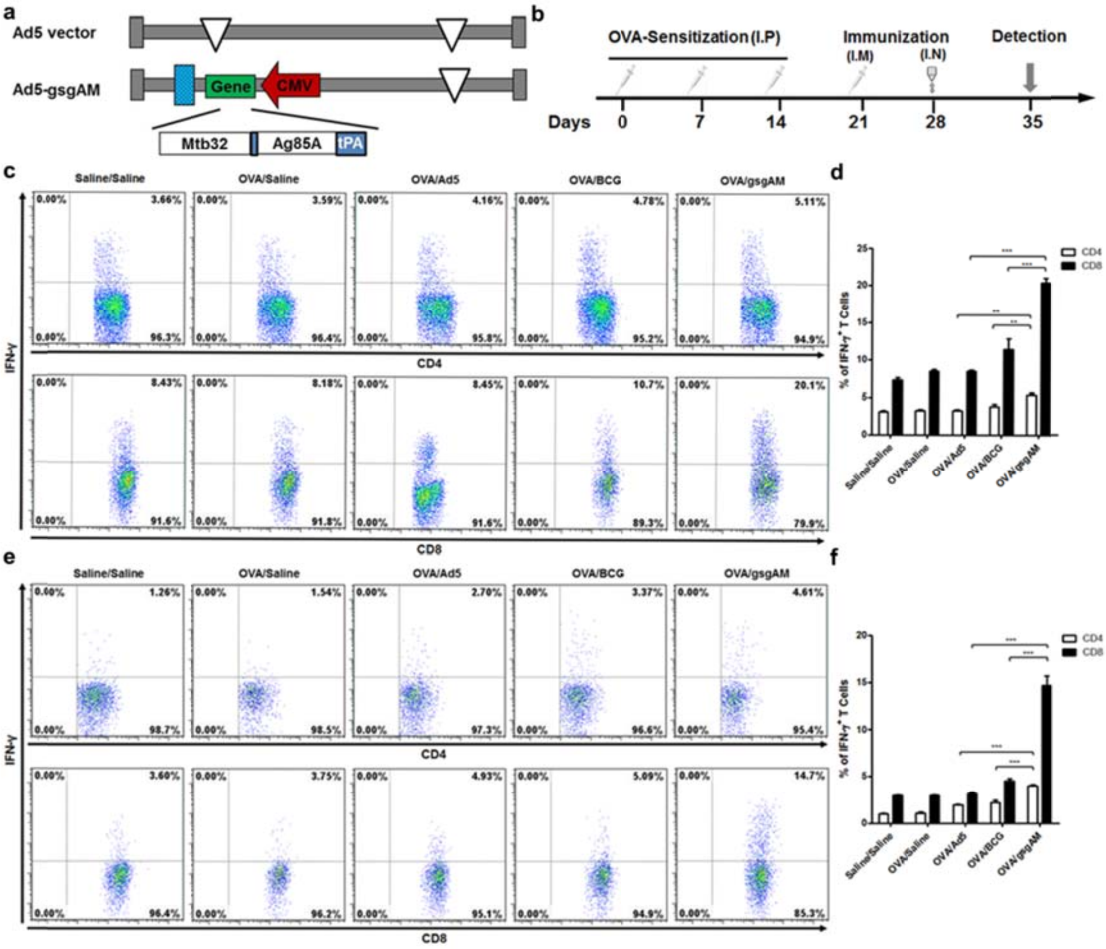

Figure S1. The immunogenicity of Ad5-gsgAM in OVA-sensitized mice. 6-week-old mice were sensitized with OVA and immunized with Ad5, BCG, or Ad5-gsgAM through a prime-boost strategy. One week after the final immunization, the spleen and lung tissues were dissected out for analysis. **a** Diagram of the genomes of Ad5 and Ad5-gsgAM. **b** Schedules of OVA sensitization, immunization and detection. **c** Representative dot plots of IFN-γ^{+}CD4^{+} (upper panel) and IFN-γ^{+}CD8^{+} (bottom panel) T cells in the splenic lymphocytes stimulated with PMA+ionomycin. **d** The percentages of IFN-γ^{+}CD4^{+} and IFN-γ^{+}CD8^{+} T

cells in total $CD4^+$ or $CD8^+$ T cells in the spleen. **e** Representative dot plots of IFN-$\gamma^+$$CD4^+$ (upper panel) and IFN-$\gamma^+$$CD8^+$ (bottom panel) T cells in the MLN lymphocytes stimulated with PMA+ionomycin. **f** The percentages of IFN-$\gamma^+$$CD4^+$ and IFN-$\gamma^+$$CD8^+$ T cells in total $CD4^+$ or $CD8^+$ T cells in the MLN. Lymphocytes were isolated from the spleens and lungs of different groups of mice and were stimulated with PMA+ionomycin for 6h. Then the cells were stained with CD3-PerCP, CD4-FITC, CD8-APC, and IFN-γ-PE, and subjected to FACS analysis. Representative results from one of three independent experiments are shown. ** $P < 0.01$, *** $P < 0.001$.
