# Supplementary material for: Immunization with an adenovirus-vectored TB vaccine containing Ag85A-Mtb32 effectively alleviates allergic asthma
Source: J Mol Med (Berl). 2018 Jan 4;96(3):249–63. doi: 10.1007/s00109-017-1614-5 (PMC5859035; doi:10.1007/s00109-017-1614-5)
Supplement: Supplementary file 2 — (PDF 158 kb) [file 109_2017_1614_MOESM2_ESM.pdf]

**Title:** Immunization with an adenovirus-vectored TB vaccine containing Ag85A-Mtb32 effectively alleviates allergic asthma

**Journal Name:** Journal of Molecular Medicine

**Authors:** Yiling Zhang[1,2,3¶], Ying Feng[1,2¶], Liang Li[1,2¶], Xianmiao Ye[2], Jinlin Wang[2], Qian Wang[2], Pingchao Li[1,2], Na Li[1], Xuehua Zheng[2], Xiang, Gao[2], Chufang Li[1], Feng Li[4], Baoqing Sun[1], Kefang Lai[1], Zhong Su[2], Nanshan Zhong[1], Ling Chen[1,2]*, and Liqiang Feng[2]*

**Affiliations:**

[1]State Key Laboratory of Respiratory Disease, The First Affiliated Hospital of Guangzhou Medical University, Guangzhou, China, [2]Guangzhou Institutes of Biomedicine and Health, Chinese Academy of Sciences, Guangzhou, China, [3]Department of Respiratory Medicine, Guizhou Provincial People's Hospital, Guiyang, China. [4]Institute of Infectious Diseases, Guangzhou Eighth people's Hospital, Guangzhou Medical University, China.

[¶]These authors contributed equally to this work.

*Correspondence should be addressed to:

L.F. (E-mail: feng_liqiang@gibh.ac.cn; Tel: +86-20-32015289; Fax: +86-20-32015299), or L.C. (E-mail: chen_ling@gibh.ac.cn; Tel: +86-20-32015289; Fax: +86-20-32015299), Guangzhou Institutes of Biomedicine and Health, Chinese Academy of Sciences, 190 Kai Yuan Avenue, Science Park, Guangzhou, China.

## Supplementary Figure S2

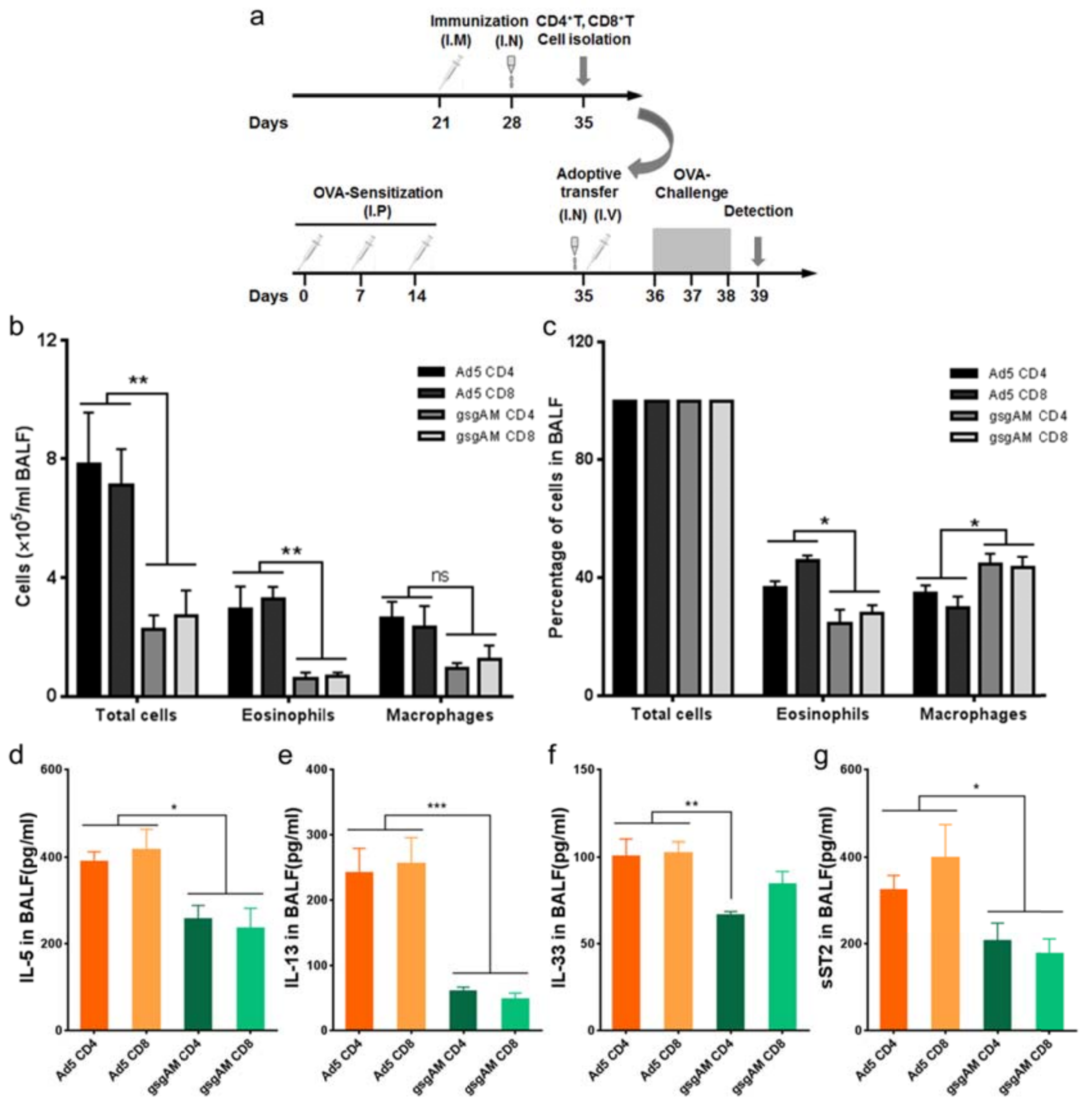


**Figure S2. Ad5-gsgAM-induced CD4$^+$T and CD8$^+$T cells showed suppression on allergic airway inflammation.** 6-week-old mice were immunized with Ad5-gsgAM or Ad5 through a prime-boost strategy. One week after the final immunization, CD4$^+$T and CD8$^+$T cells were isolated from the spleens and lungs. Another four groups of mice were sensitized with OVA. One day before challenge, OVA-sensitized mice were inoculated with CD4$^+$T or CD8$^+$T cells. One day after challenge, mice were euthanatized and examined. **a** Schedules of immunization, T cell

isolation, adoptive transfer and detection. **b** The absolute numbers of total cells, eosinophils and macrophages in the BALFs were counted based on H&E staining. **c** The percentages of total cells, eosinophils and macrophages in the BALFs. **d-g** The contents of IL-5 (**d**), IL-13 (**e**), IL-33 (**f**), and sST2 (**g**) in the BALFs were determined by ELISA. Data are presented as the mean ± SEM (n = 5 mice per group). Representative results from one of two independent experiments are shown. * $P < 0.05$, ** $P < 0.01$, *** $P < 0.001$. ns, no significance.
