# Supplementary material for: Immunization with an adenovirus-vectored TB vaccine containing Ag85A-Mtb32 effectively alleviates allergic asthma
Source: J Mol Med (Berl). 2018 Jan 4;96(3):249–63. doi: 10.1007/s00109-017-1614-5 (PMC5859035; doi:10.1007/s00109-017-1614-5)
Supplement: Supplementary file 3 — (PDF 112 kb) [file 109_2017_1614_MOESM3_ESM.pdf]

Title: Immunization with an adenovirus-vectored TB vaccine containing Ag85A-Mtb32 effectively alleviates allergic asthma

Journal Name: Journal of Molecular Medicine

Authors: Yiling Zhang[1,2,3¶], Ying Feng[1,2¶], Liang Li[1,2¶], Xianmiao Ye[2], Jinlin Wang[2], Qian Wang[2], Pingchao Li[1,2], Na Li[1], Xuehua Zheng[2], Xiang, Gao[2], Chufang Li[1], Feng Li[4], Baoqing Sun[1], Kefang Lai[1], Zhong Su[2], Nanshan Zhong[1], Ling Chen[1,2]*, and Liqiang Feng[2]*

Affiliations:

[1]State Key Laboratory of Respiratory Disease, The First Affiliated Hospital of Guangzhou Medical University, Guangzhou, China, [2]Guangzhou Institutes of Biomedicine and Health, Chinese Academy of Sciences, Guangzhou, China, [3]Department of Respiratory Medicine, Guizhou Provincial People's Hospital, Guiyang, China. [4]Institute of Infectious Diseases, Guangzhou Eighth people's Hospital, Guangzhou Medical University, China.

[¶]These authors contributed equally to this work.

*Correspondence should be addressed to:

L.F. (E-mail: feng_liqiang@gibh.ac.cn; Tel: +86-20-32015289; Fax: +86-20-32015299), or L.C. (E-mail: chen_ling@gibh.ac.cn; Tel: +86-20-32015289; Fax: +86-20-32015299), Guangzhou Institutes of Biomedicine and Health, Chinese Academy of Sciences, 190 Kai Yuan Avenue, Science Park, Guangzhou, China.

Supplementary Figure S3

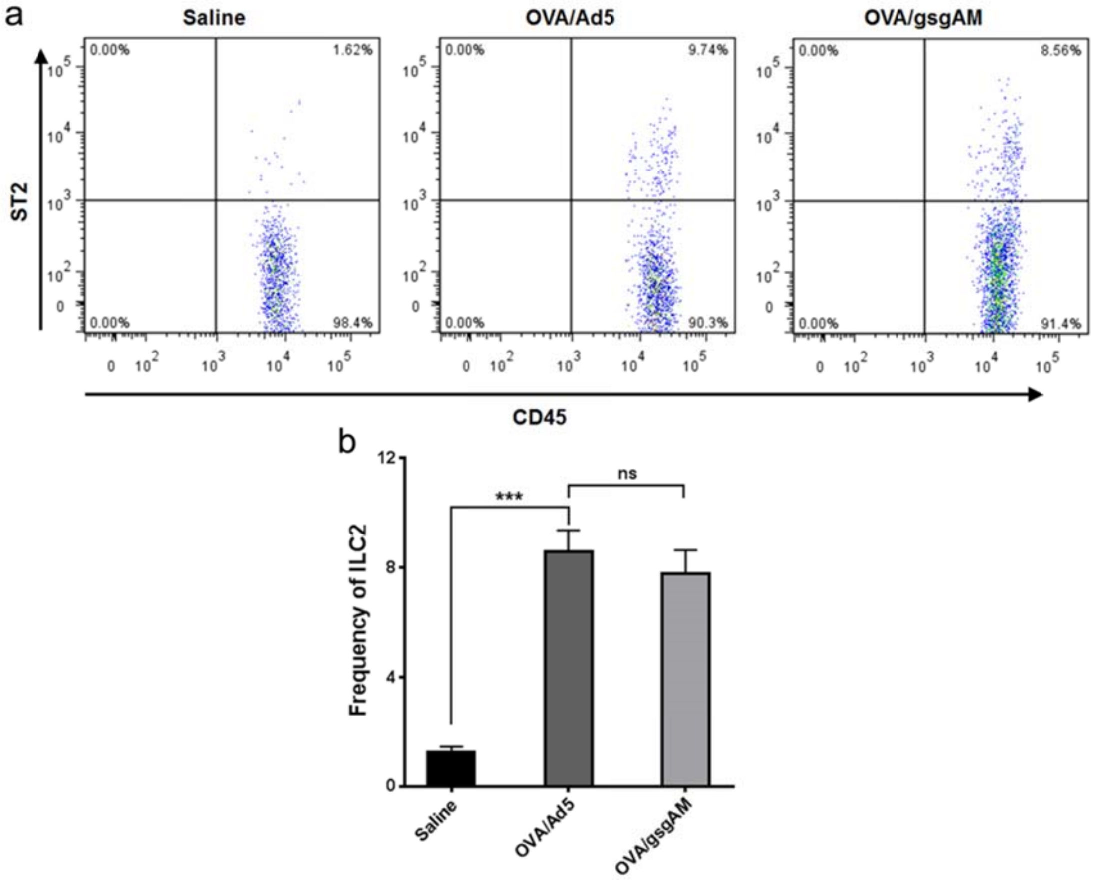

Figure S3. Ad5-gsgAM immunization did not significantly inhibit ILC2 recruitment. Lymphocytes were isolated from the lungs of Saline, OVA/Ad5 and OVA/gsgAM mice. The lymphocytes were stained with lineage antibodies (CD3, CD4, CD8, CD11c, CD19, FceRIa, Gr-1, NK1.1, Ter-199) and antibodies to CD25, CD45 as well as ST2, and then analyzed using flow cytometry. Lineage$^-$CD25$^+$CD45$^+$ST2$^+$ cells were designated as ILC2. **a** Representative dot plots of ILC2 from each group of mice. **b** The frequency of ILC2s in Lineage$^-$CD25$^+$ lung lymphocytes. Data are presented as the mean ± SEM (n = 5 mice per group).

Representative results from one of two independent experiments are shown. *** $P < 0.001$. ns, no significance.
