# Supplementary material for: Immunization with an adenovirus-vectored TB vaccine containing Ag85A-Mtb32 effectively alleviates allergic asthma
Source: J Mol Med (Berl). 2018 Jan 4;96(3):249–63. doi: 10.1007/s00109-017-1614-5 (PMC5859035; doi:10.1007/s00109-017-1614-5)
Supplement: Supplementary file 4 — (PDF 130 kb) [file 109_2017_1614_MOESM4_ESM.pdf]

Title: Immunization with an adenovirus-vectored TB vaccine containing Ag85A-Mtb32 effectively alleviates allergic asthma

Journal Name: Journal of Molecular Medicine

Authors: Yiling Zhang[1,2,3¶], Ying Feng[1,2¶], Liang Li[1,2¶], Xianmiao Ye[2], Jinlin Wang[2], Qian Wang[2], Pingchao Li[1,2], Na Li[1], Xuehua Zheng[2], Xiang, Gao[2], Chufang Li[1], Feng Li[4], Baoqing Sun[1], Kefang Lai[1], Zhong Su[2], Nanshan Zhong[1], Ling Chen[1,2]*, and Liqiang Feng[2]*

Affiliations:

[1]State Key Laboratory of Respiratory Disease, The First Affiliated Hospital of Guangzhou Medical University, Guangzhou, China, [2]Guangzhou Institutes of Biomedicine and Health, Chinese Academy of Sciences, Guangzhou, China, [3]Department of Respiratory Medicine, Guizhou Provincial People's Hospital, Guiyang, China. [4]Institute of Infectious Diseases, Guangzhou Eighth people's Hospital, Guangzhou Medical University, China.

[¶]These authors contributed equally to this work.

*Correspondence should be addressed to:

L.F. (E-mail: feng_liqiang@gibh.ac.cn; Tel: +86-20-32015289; Fax: +86-20-32015299), or L.C. (E-mail: chen_ling@gibh.ac.cn; Tel: +86-20-32015289; Fax: +86-20-32015299), Guangzhou Institutes of Biomedicine and Health, Chinese Academy of Sciences, 190 Kai Yuan Avenue, Science Park, Guangzhou, China.

Supplementary Materials and Methods.

Airway hyper-responsiveness

One day after the final challenge, the AHR to inhaled methacholine (MCh) was measured using a single-chamber, whole-body plethysmography. In brief, the baseline values were recorded, and then the mice were successively exposed to PBS or doubled concentrations of MCh starting from 6.25 to 200mg/ml. At each concentration, the MCh aerosolization and incubation were each performed for 1min, and the animals were stimulated for 3min. Finally, the enhanced pause (Penh) was recorded and averaged for 3min. The results were normalized by subtracting the baseline values.

Bronchoalveolar lavage, cell counting and histological examination

One day after the final challenge, the bronchoalveolar lavage fluids (BALFs) were collected. In brief, the lungs were repeatedly lavaged (3 times) with 0.8ml of chilled PBS. The recovered BALFs were centrifuged, and the cell pellets were re-suspended in PBS，counted by trypan blue dye exclusion，and then mounted onto slides. The monocytes, eosinophils, lymphocytes, and neutrophils were counted in a blinded manner using hematoxylin-eosin (H&E) staining. The cell-free BALFs were stored at -80°C for subsequent analysis.

The lung tissues were harvested and fixed overnight in 4% paraformaldehyde. After dehydration, the tissues were embedded, sectioned and stained with H&E or periodic acid-Schiff (PAS). The histological and PAS scores were determined in a blinded manner as follows: A. peribronchiolar infiltration (0, normal; 1, ≤25%; 2, 25-75%; 3, >75%); B. qualitative analysis (0, normal; 1, ≤3 cells thick in diameter; 2, 3–10 cells thick; 3, >10 cells thick); C. bronchiolar leakage (0, normal; 1, ≤ 25%; 2, >25%); D. perivascular infiltration (0, normal; 1, ≤10%; 2, 10-50%; 3, >50%); E. parenchymatous pneumonia (0, normal; 1, mild to moderate; 2, severe). The final scores were calculated as A+3 × (B+C)+D+E. The PAS scores were calculated according to the percentage of PAS-stained goblet cells versus the epithelial cells in the airway (0, <5%; 1, 5–25%; 2, 25–50%; 3, 50–75%; and 4, >75% goblet cells). At least 5 random fields in each slide were counted, and the average of the airway scores were calculated (n=5 mice per group).

ELISpot assay

To detect the IFN-γ and IL-4 releasing cells, 96-well plates (Merck Millipore, Billerica, MA) were activated and coated with anti-mouse IFN-γ or IL-4 antibody (BD Biosciences, San Diego, CA) overnight at 4°C. The lymphocytes from spleens and mediastinal lymph nodes (MLN) were isolated and seeded into the plates with the addition of OVA

(10μg/ml) or ConA (10ug/ml). After incubating for 24h for IFN-γ detection and 48h for IL-4 detection, the plates were developed with biotinylated anti-mouse IFN-γ or IL-4 antibodies (BD Biosciences, San Diego, CA) and NBT/BCIP reagent (Pierce, Rockford, IL). Finally, the IFN-γ or IL-4 releasing cells were counted by a plate reader (Bioreader 4000, BIOSYS, Germany).

Quantification of iNOS and iNOS-expressing cells

Lung tissues were harvested, homogenized and centrifuged at 12000rpm at 4℃. The supernatants were collected and subjected to SDS-PAGE. The separated proteins were transferred onto nitrocellulose membranes and probed with monoclonal anti-iNOS or anti-β-actin antibodies (Santa Cruz, Dallas, TX). The membrane was developed with a goat-anti-mouse IgG and detected using an ECL kit (Merck Millipore, Billerica, MA). The gel graphs were quantitatively analyzed using ImageJ software (NIH, Bethesda, MD). The density of each bands were read and the net band values were obtained by deducting the background value. The relative band value was then calculated as the ratio of the net band value of iNOS vs that of β-actin.

iNOS-expressing cells in the lung tissue sections were assessed as follows: Images of immuno-stained lung tissue sections from each group of mice (n = 4 to 6 mice per group) were randomly obtained by light

microscopy at 200× magnification. Four images of each mouse were counted for iNOS-positive cells in a blinded manner, and the average of the number of iNOS-positive cells in one square millimeter was calculated.

T cell adoptive transfer assay

6-week-old female C57/BL6 mice were sensitized with OVA. Another age-matched mice were immunized with Ad5-gsgAM (2.5×10^{9} viral particles per mouse) or Ad5 empty vector (2.5×10^{9} viral particles per mouse) by a I.M priming plus I.N boost strategy at one-week interval. One week after the second immunization, T lymphocytes were isolated from the spleens and lungs. $CD4^{+}$T and $CD8^{+}$T cells were collected using $CD4^{+}$T Cell Isolation Kit and $CD4^{+}$T Cell Isolation Kit (Miltenyi Biotec, Auburn, CA) according to manufactory's instructions, respectively. After purification by negative selection, splenic and lung T cells were injected into OVA-sensitized recipient mice through intravenous and intranasal routes, respectively. Each recipient mice received a total of 5×10^{6} splenic T cells and 7×10^{5} lung T cells. One day after T cell adoptive transfer, recipient mice were challenged with OVA for 3 consecutive days. One day after challenge, mice were sacrificed and the BALFs were collected and subjected to analysis.

Analysis of group 2 innate lymphoid cells (ILCs)

To analyze the ILC2s in the lungs, lymphocytes were isolated from the lungs of Saline, OVA/Ad5 and OVA/gsgAM mice. 5×10^6 lymphocytes were stained with lineage antibodies including CD3-eFluor 450, CD4-eFluor 450, CD11c-eFluor 450, CD19-eFluor 450, FceRIa-eFluor 450, Gr-1-eFluor 450, NK1.1-eFluor 450, Ter-119-eFluor 450 (ThermoFisher Scientific, Waltham, MA), CD8-PE (BD Biosciences, San Diego, CA), CD25-APC, CD45-FITC (ThermoFisher Scientific, Waltham, MA), and ST2-biotin (Biolegend, San Diego, CA). After incubation for 30min at 4℃, the cells were washed with PBS and stained with APC/Cy7 labelled streptavidin. Finally, the cells were washed and analyzed with a FACSAria flow cytometer (BD Biosciences, San Diego, CA).
